# Intracranial Hemorrhage Secondary to Newly Diagnosed Acute Promyelocytic Leukemia: A Cautionary Tale

**DOI:** 10.7759/cureus.23252

**Published:** 2022-03-17

**Authors:** Kanksha Peddi, Brandon Wiggins, Omar Choudhury, Casey Reulbach, Paul Adams

**Affiliations:** 1 Internal Medicine, Ascension Genesys Hospital, Grand Blanc, USA; 2 Neurology, Henry Ford Health System, Detroit, USA; 3 Hematology-Oncology, Ascension Genesys Hospital, Grand Blanc, USA

**Keywords:** acute promyelocytic leukemia, neuro oncology, neurology, intracranial hemorrhage, ich, apl, hematology-oncology

## Abstract

Acute promyelocytic leukemia (APL) typically presents with complications from pancytopenia, generalized weakness, and hemorrhagic findings, with a distinguishing feature being the associated predilection of disseminated intravascular coagulation (DIC). APL is characterized by the halting of cellular differentiation in the promyelocyte stage, and balanced chromosomal translocation t(15;17) (q24;q21) that forms the promyelocytic leukemia-retinoic acid receptor-α (PML-RARA) fusion protein present in 95% of cases. APL has a high rate of early mortality secondary to coagulopathy, lending to the imperative need to begin a differentiation agent as soon as the disease is suspected, with all-trans retinoic acid (ATRA) being the most common differentiation agent. Herein, we present the case of a 32-year-old man presenting with non-specific symptoms of fatigue and scattered bruising, who was found to have an intracranial hemorrhage (ICH) in the setting of suspected APL. This case illuminates the importance of early brain imaging in suspected cases of APL to conceivably lessen the severity of hemorrhagic complications and represents a cautionary tale for similar cases in the future.

## Introduction

Acute promyelocytic leukemia (APL) accounts for 10%-15% of acute myeloid leukemia (AML) with the highest incidence occurring in early adulthood, without gender predilection. This cancer is characterized by the cessation of cellular differentiation at the promyelocyte stage, and typically carries the chromosomal translation t(15;17) (q24;q21) [1]. Patients generally present with symptoms related to pancytopenias such as weakness, fatigability, infections, and hemorrhagic complications such as gingival bleeding, epistaxis, menorrhagia, or bruising [2]. The suspected diagnosis of APL is confirmed through bone marrow biopsy and fluorescence in-situ hybridization (FISH) genetic testing. If left untreated, the prognosis is poor, with a median survival of less than one month [3]. However, APL has an excellent prognosis if caught early, before catastrophic complications such as intracranial hemorrhage (ICH) can occur. Patients at low risk (white blood cell (WBC) < 10,000/µL and platelets > 40,000/µL) at time of diagnosis have a relapse-free survival (RFS) at 3 years of 98%, and patients in a high-risk category (WBC > 10,000/µL) have an RFS of 70% at 3 years after treatment with arsenic trioxide and all-trans retinoic acid (ATRA) [4]. Here, we describe a case of a young man in early adulthood who presented with vague symptoms and had life-threatening complications of APL.

## Case presentation

A previously healthy 32-year-old male with no past medical history was evaluated for complaints of easy bruising, fatigue, and weakness for three weeks. Most notably on presentation, he complained of incredible fatigue resulting in an excess of sleep upwards of 15 hours a day. He denied fevers, chills, cough, abdominal pain, falls, trauma, or any other complaints. The patient saw his primary care physician over video call the same day who recommended that he be evaluated in the Emergency Department.

Upon arrival, he was found to be hypertensive at 164/72 mmHg, heart rate of 94, respiratory rate of 18 breaths per minute, afebrile at 98.3°F, and on room air with an oxygen saturation of 97%. Physical exam on the day of evaluation revealed scattered petechiae over the lower extremities along with purpuric rash over the abdomen and axillae. There were also several large areas of ecchymosis over the bilateral thighs and arms. In addition, palatal petechiae were noted on examination of the oral mucosa. The patient had no focal neurological deficits on the exam and was moving all of his extremities with 5/5 strength and had fluent speech. The remainder of the exam was unremarkable.

**Figure 1:**
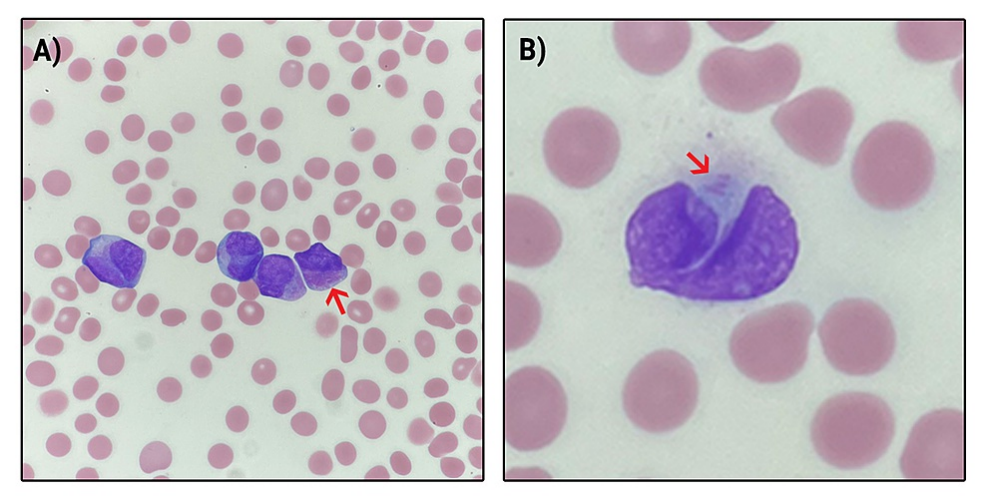
Auer rods A) Patient’s bone marrow biopsy featuring Auer rods classic for APL; B) magnified view of the Auer rods.

Initial laboratory findings were significant for a WBC of 66.5 k/µL, hemoglobin (Hb) of 10.3 g/dL, platelet count of 13 k/cmm, hematocrit (Hct) of 28.7%, mean corpuscular volume (MCV) of 87.9 Fl, absolute neutrophil count (ANC) of 28.6 k/µL, absolute lymphocyte count (ALC) of 17.2 k/µL. The patient’s chemistry panel showed sodium of 141 mmol/L, potassium of 3.5 mmol/L, bicarbonate of 25 mmol/L, blood urea nitrogen of 19 mg/dL, creatinine level of 1.07 mg/dL, and lactate dehydrogenase at 1076 IU/L. Initial findings were significant for a large number of blasts at 29%, metamyelocytes at 9%, myelocytes at 15%, and promyelocytes at 9% (Table 1). Peripheral slide morphology prepared from admission showed the presence of Auer rods (Figure 1). The hematology/oncology service was consulted for further management. Coagulation panel studies were not performed until day two of admission but were significant for prolonged prothrombin time (PT) of 14.9 sec; prolonged international normalized ratio (INR) of 1.32; fibrinogen within normal limits of 246 mg/dL, a decreased partial thromboplastin time (PTT) of 25 sec (Table 1). A respiratory viral panel including coronavirus disease-2019 (COVID-19) PCR testing was negative.

**Table 1:** Initial laboratory findings

Laboratory Values	Measured	Normal Range
White blood cell count (WBC)	66.5 k/µL	4.5 - 11.0 k/µL
Platelet Count (Plt)	13 k/cmm	140 - 440 k/cmm
Hemoglobin (Hb)	10.3 g/dL	11.0 - 16.2 g/dL
Hematocrit (Hct)	28.7%	36% - 46%
Mean corpuscular volume (MCV)	87.9 Fl	80.0 - 100.0 Fl
Absolute neutrophil count (ANC)	28.6 k/cmm	1.0 - 8.0 k/cmm
Absolute lymphocyte count (ALC)	17.2 k/µL	1.0 - 5.0 k/cmm
Sodium (Na)	141 mmol/L	136 - 144 mmol/L
Potassium (K+)	3.5 mmol/L	3.6 - 5.1 mmol/L
Bicarbonate (HCO3^−^)	25 mmol/L	20 - 30 mmol/L
Blood urea nitrogen (BUN)	19 mg/dL	8 - 26 mg/dL
Creatinine (Cr)	1.07 mg/dL	0.61 - 1.24 mg/dL
Lactate dehydrogenase (LDH)	1076 IU/L	125 - 220 IU/L
Blasts	29 %	0 - 0 %
Metamyelocytes	9 %	0 - 0 %
Myelocytes	15%	0 - 0 %
Promyelocytes	9%	0 - 0 %
Prothrombin time (PT)	14.9 sec	10.1 - 12.5 sec
Partial thromboplastin time (PTT)	25 sec	26 - 39 sec
International normalized ratio (INR)	1.32	0.80 - 1.2
Fibrinogen	246 g/dL	202 - 597 mg/dL

The patient was admitted to the intensive care unit (ICU) for suspicion of leukemia. He was treated with broad-spectrum antibiotics and intravenous fluid resuscitation. His weakness at this point was suspected likely related to acute leukemia. Subsequent WBC count was elevated at 73.5 k/µL, with 44% blasts on the differential. Platelet count showed worsening thrombocytopenia at 12 k/Cmm. The patient underwent a bone marrow biopsy approximately two days after the presentation which suggested acute promyelocytic lymphoma based on classic Auer rods seen in Figure 1. While awaiting confirmation, the patient’s WBC count rose to 107,000 k/µL with 73% blasts on the differential, along with a platelet count of 82 k/Cmm.

**Figure 2:**
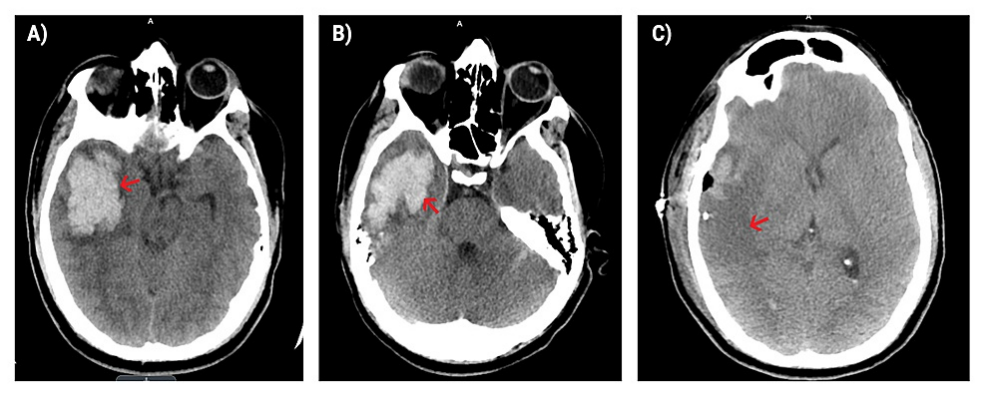
Intracerebral hemorrhage A) Intraparenchymal hemorrhage visualized in the right frontotemporal region; B) prominent right temporal intracerebral hemorrhage; C) evolution of the right hemispheric intracerebral hemorrhage now showing post-craniotomy changes with slight midline shift.

Despite the mild improvement of thrombocytopenia, the patient began complaining of a severe 10/10 headache, for which a non-contrast computed tomography (CT) of the head was acquired. CT of the head revealed a large approximately 4 cm intraparenchymal hemorrhage involving the right anterior temporal lobe with surrounding edema resulting in a medial displacement of the right uncus and partial effacement of the right lateral ventricle (Figure 2A). Neurosurgery was consulted and recommended non-surgical intervention. They suggested frequent neuro checks, supportive care, and strict blood pressure management. The following day, a similar appearance of the large right temporal hemorrhage was noted but it was now associated with mass effect and moderate midline shift.

**Figure 3:**
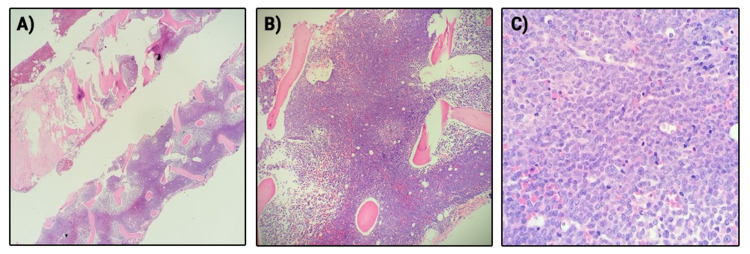
Bone marrow biopsy (H&E Stain) Bone marrow biopsy with hematoxylin and eosin (H&E) stain showing hypercellularity, A) 20× magnification; B) 100× magnification; C) 400× magnification.

**Figure 4:**
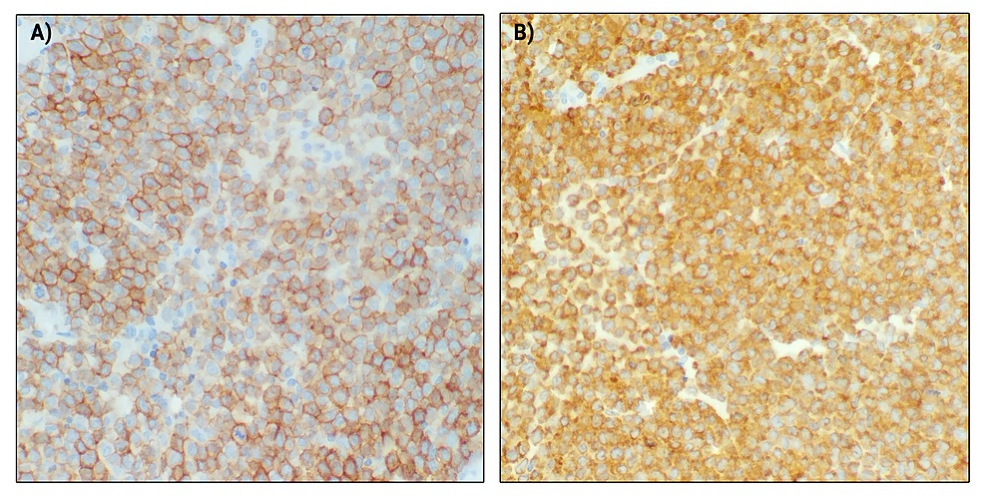
CD117 & MPO stain A) CD117 stain and B) myeloperoxidase (MPO) stain of bone marrow, markers of immaturity, showing hypercellular proliferation of leukemia.

The patient underwent leukapheresis and was treated with oral hydroxyurea 2 g every 8 hours and received transfusions. Anticoagulation and antiplatelet agents were held. Bone marrow biopsy (Figures 3-4) resulted in a diagnosis of APL and ATRA was initiated with a dose of 50 mg twice daily. The patient was also continued on hydroxyurea 2 g three times daily. After awaiting confirmation of acute progranulocytic leukemia with promyelocytic leukemia (PML)/retinoic acid receptor-α (RARA) gene transfusion by FISH, it was confirmed that both bone marrow biopsy and flow were consistent with APL. The patient was confirmed to be t(15;17) positive.

Close monitoring for tumor lysis syndrome and other fatal complications were initiated due to the patient’s increased risk of disseminated intravascular coagulation (DIC). Chemotherapy was initiated with idarubicin 18 mg on days 2, 4, 6, and 8. Arsenic therapy 0.15 mg/kg IV on days 9-36 was planned. Blast count continued to rise and platelets were monitored and maintained at approximately 100 K in order to prevent rebleeding. The patient developed dysarthria, left hemiparesis, neglect, and repeat CT head showed prominent parenchymal hemorrhage in the right temporal lobe with worsening mass effect and an 8 mm leftward midline shift (Figure 2B). The patient had multiple transfusions throughout the remainder of the day along with elevations in both WBC and blast count but ultimately became hemodynamically unstable, requiring intubation and sedation for airway protection. Due to worsening hemodynamic status and increased bleeding, he was deemed an emergent surgical candidate and was taken to the operating room where he underwent a right temporal craniotomy for evacuation of the hematoma along with placement of an intracranial pressure monitor. Postoperative changes on CT confirmed the evacuation of a significant amount of hemorrhage that was present in the R hemisphere and improvement in the size of the left lateral ventricle along with an improvement in midline shift (Figure 2C). The patient was also placed on dexamethasone 20 mg twice daily by hematology/oncology to prevent differentiation syndrome from ATRA. For the remaining week, the patient was intubated, sedated, and paralyzed in the ICU until becoming hemodynamically stable. The patient later improved and underwent tracheostomy and percutaneous endoscopic gastrostomy (PEG) placement.

A repeat bone marrow biopsy was performed after induction chemotherapy and he was maintained on ATRA. His white counts trended down to 13 k/µL, hemoglobin was 7.8 g/dL, and platelet count improved at 238 k/Cmm. His repeat biopsy showed no aberrant blasts on flow cytometry. His repeat peripheral smear was consistent with a reactive marrow and not an acute leukemia appearance and without an increase in promyelocytes. He is scheduled to undergo consolidation chemotherapy. Though hematologically and neurologically, the patient has improved, he still remains in the ICU for undulating pressures, hemodynamic instability, and risk for fatal complications.

## Discussion

APL has a mean incidence of 700 cases per year and accounts for approximately 5 to 20% of AML cases in the United States [5]. APL is seen in all ethnicities in the US, with higher rates among Latinos than Caucasians and Asians with African Americans having the least lifetime likelihood of developing the disease [6]. Incidence over the last two decades has increased due to unknown factors, but likely due to improved testing, healthcare accessibility, APL awareness, and surveillance [7]. APL differs from AML in the age of diagnosis, as it is most common in teenagers and young adults, but can also be seen in people up until their 6th decade and then rates decrease thereafter.

APL is in the family of AML, as it is a neoplasm of the myeloid lineage affecting specifically promyelocytes. APL is distinctive from AML as it affects promyelocytes and it almost always involves a translocation on the 17th chromosome, with involvement of the RARA [8]. Retinoic acid functions as a tissue differentiator for multiple tissues in the human body and in this instance, specifically in myeloid cells [9]. Around 92% of APL cases are related to the PML. When overexpressed, PML inhibits growth in cell lines and in concert with RARA dysfunction, leads to non-differentiation of promyelocytes to mature neutrophils or macrophages and proliferation that is seen in APL [10]. APL is distinguished by having atypical promyelocytes in peripheral blood that are especially large, usually over 20µ, and significant for violet granules in the cytoplasm that conceal the cell’s nucleus [11].

Two forms of APL pre-dominate and differ from normal promyelocytes in their immunophenotype, assisting in differentiation between normal and leukemic cells. The hypergranular variant expresses CD13, CD33, MPO, low levels of CD15, and CD117, and typically does not express CD34, CD11B, or histocompatibility leukocyte antigens (HLA)-DR. As opposed to the hypergranular variant, hypogranular variant express MPO, CD2, and CD34 with HLA-DR negative [12].

Patients usually present with symptoms related to pancytopenia including fatigue, chest pain, palpitations, shortness of breath, bleeding, bruising, and recurrent infections. Additionally, patients that have been on a topoisomerase inhibitor for previous cancer treatment, such as lymphoma, breast cancer, multiple sclerosis, or received radiation can iatrogenically acquire APL [13]. Patients with APL are at an extremely high risk of both bleeding and clotting due to their hematologic malignancy and DIC and primary hyperfibrinolysis are often common [14].

Pertinent to this case, primary hyperfibrinolysis caused by APL is the culprit to pulmonary or cerebral hemorrhage in extreme circumstances that may lead to death [15]. Tissue factor is activated in APL due to abnormal RARA leading to increased coagulation. Apoptosis of APL cells also encourages coagulation through increased thrombin, plasmin, and fibrin production [16]. Primary hyperfibrinolysis, on the other hand, leads to an extreme anti-coagulant state caused by annexin II activating tissue plasminogen activator (tPA), which is overexpressed in APL, likely the culprit for intracerebral hemorrhage in this case of an otherwise healthy patient [17].

APL can be suspected in patients with any cytopenia or pancytopenia, especially those with leukopenia, and suspicious in otherwise healthy patients that have severe coagulopathy with otherwise unknown cause. Making the diagnosis for APL can be aided by peripheral smear and/or bone marrow biopsy, where immature and leukemic myeloid cells will be identified, as well as the aforementioned immunohistochemistry. Definitive diagnosis is made by genetic testing for chromosomal translocation of 15;17 and/or PML-RARA fusion gene via conventional karyotyping, FISH, or PCR [18].

Induction treatment is based on the determined risk of APL, with WBC counts over 10,000/µL and platelets less than 40,000/µL being considered high risk and white blood cells less than 10,000/µL and platelets greater than 40,000/µL considered low risk. Low-risk APL induction therapy usually consists of ATRA in combination with arsenic trioxide. High-risk APL induction therapy differs in the sense that ATRA is usually combined with anthracycline-based chemotherapy to prevent extreme leukocytosis and the implications that result from this phenomenon [19].

The prognosis of APL depends on multiple factors including blood cell counts at diagnosis, age, and complications experienced but overall has very good mortality and morbidity outcomes if treated promptly. This includes and warrants the use of prompt brain imaging when suspicion of APL is high. Low-risk APL has a three-year RFS of 98%, whereas high-risk APL has a three-year RFS of 70% [20]. Patients that are younger, typically fare better than patients over 60, but APL has better rates of survival than AML in this same age group [3].

## Conclusions

This paper presents the case of a 32-year-old man who presented to the Emergency Department with complaints of fatigue and generalized weakness, found to have petechial rashes and blood count abnormalities including anemia, leukocytosis, and thrombocytopenia. The patient’s course was complicated by intracerebral hemorrhage, which was not elucidated until two days after presentation. This case highlights the importance of acquiring early brain imaging in suspected leukemia patients despite a lack of clear focal neurological abnormalities. Additional research into the incidence of intracerebral hemorrhage in leukemia patients with unclear neurological deficits is imperative in order to potentially decrease fatal complications from this devastating condition.
